# A Preliminary Study on the Effect of Psyllium Husk Ethanolic Extract on Hyperlipidemia, Hyperglycemia, and Oxidative Stress Induced by Triton X-100 Injection in Rats

**DOI:** 10.3390/biology10040335

**Published:** 2021-04-16

**Authors:** Mohamed A. Hashem, Noura A. Abd-Allah, Essam A. Mahmoud, Shimaa A. Amer, Mohamed Alkafafy

**Affiliations:** 1 Clinical Pathology Department, Faculty of Veterinary Medicine, Zagazig University, Alzeraa Street, Zagazig 44511, Egypt; mhashem.vet@gmail.com (M.A.H.); omarfahd334@gmail.com (N.A.A.-A.); essammahmoud97@yahoo.com (E.A.M.); 2 Department of Nutrition & Clinical Nutrition, Faculty of Veterinary Medicine, Zagazig University, Zagazig 44511, Egypt; 3 Department of Biotechnology, College of Science, Taif University, P.O. Box 11099, Taif 21944, Saudi Arabia; m.kafafy@tu.edu.sa

**Keywords:** hyperlipidemia, psyllium husk ethanolic extract, Triton X-100, leptin, antioxidants

## Abstract

**Simple Summary:**

Hyperlipidemia contributes to the emergence and development of circulatory diseases, which commonly causes morbidity and mortality. The present study evaluated the psyllium husk ethanolic extract (PHEE) effect on hyperlipidemia induced by Triton X-100. This study proved that Triton-induced hyperlipidemic rats with PHEE administration reduced hyperlipidemia by hepatic and pancreatic protective effects. The administration of PHEE lowered lipid peroxidation, increased antioxidant activity, and improved leptin and adiponectin hormone levels.

**Abstract:**

The aim of this study is to assess the efficiency of psyllium husk ethanolic extract (PHEE) on Triton X-100 induced hyperlipidemic rats by studying the changes in hepatic and pancreatic function and histopathology. Forty male albino rats (bodyweight 175–188 g) were grouped randomly into four sets with ten rats. The experimental groups included: (1) control group (CON); (2) Triton X-100 induced hyperlipidemic group—rats were intraperitoneally injected with a single dose of Triton X-100 (100 mg/kg body weight) on the 21st day of Trial onset; (3) PHEE group—PHEE was orally administered (100 mg/kg body weight dissolved in 1 mL of distilled water) by gastric tube from the first day of the experiment until the fortieth day, once daily, (PHEE); (4) PHEE +Triton group, which received PHEE orally with the induction of hyperlipidemia. Treating hyperlipidemic rats with PHEE showed a decrease in the total serum lipids, triglyceride (TG), total cholesterol (TC), atherogenic index (AI), and malondialdehyde (MDA) with an increase in superoxide dismutase (SOD) and catalase (CAT) activities. PHEE administration alleviated the negative impact of Triton on the serum levels of glucose, insulin, glycated hemoglobin (HbA1c), homeostatic model assessment for insulin resistance (HOMA IR index), leptin hormone, Alanine Aminotransferase (ALT), Aspartate Aminotransferase (AST), Gamma-Glutamyl Transferase (GGT) and proteinogram. The Triton-induced hyperlipidemic rats showed extensive histopathological changes in the liver and pancreas, which were alleviated with PHEE administration. It could be concluded that PHEE has potent effects against hyperlipidemia, hyperglycemia, and oxidative stress due to its biologically active constituents detected by GC-MS analysis. This study’s findings may help develop a novel trial against the effects of hyperlipidemia in the future.

## 1. Introduction

Hyperlipidemia is a major problem leading to obesity, stroke, myocardial infarction, atherosclerosis, type 2 diabetes, degenerative joint disease, and circulatory disease, the most prevalent reason for morbidity and mortality worldwide [1]. It is manifested as hypercholesterolemia and/or hypertriglyceridemia [2]. The main risk factors for cardiovascular disease include sedentary lifestyle, diet, and associated disorders such as high blood pressure, diabetes, dyslipidemia, and obesity.

Different experimental animal models in which hyperlipidemia can be induced include Triton X-100-induced, Triton WR-1339-induced, cholesterol-induced, poloxamer 407-induced, and methionine-induced hyperlipidemia. Triton X-100-induced hyperlipidemia is a recognized model [3]. Triton X-100 is a nonionic surfactant, which increases cholesterol synthesis in the liver and lipid absorption from the intestine through emulsification [4]. It inhibits lipoprotein lipase action and prevents lipoproteins’ absorption from the blood flow by the extrahepatic tissues, leading to elevated blood lipid concentrations [5]. Hyperlipidemia control involves diet regime, exercise, and hypolipidemic agents’ administration. Treating hyperlipidemia without any side effects remains a challenge in the medical field, along with drugs that are ineffective for all lipoprotein disorders. Thus, there is a need for the improvement of antihyperlipidemic therapies. Recently, attention has been drawn to medicinal plants’ use to control hyperlipidemia as an effective and safe future agent for treating hypolipidemia with few adverse effects [6] and various pharmacological properties such as antioxidant activity. The natural antioxidants present in many herbs and plants go beyond synthetic materials due to their high safety and low health risks [7,8].

Psyllium husk (*Plantago ovata*) is a dietary fiber rich in soluble ingredients and harmless and well-tolerated when used as an assistant in a low-fat diet. It also produces uncertain effects in reducing body weight. Clinical trials and meta-analysis results heartened physicians to endorse its use with dietary supplements [9]. Psyllium has numerous beneficial effects such as antidiabetic, soothing inflammation, laxative. It can aid in weight management and increasing energy [10]. Therefore, this study investigated the effect of PHEE in alleviating hyperlipidemia induced by Triton X-100 in rats.

## 2. Materials and Methods

### 2.1. Psyllium Husk Extract Preparation

Psyllium husk was obtained from the local markets for medicinal Plants, Cairo, Egypt, and accredited by the Department of Botany, Faculty of Agriculture, Zagazig University, Egypt. The psyllium husks were dried at 55 °C, ground to a fine powder, and subjected to extraction procedures, according to Nofal et al. [11]. The powder (250 g) was extracted in 1L 70% ethanol in dark-colored bottles by maceration (36 h). The resulting suspension was filtered through Whatman no. 1 filter paper, and the solvents were removed by a rotary evaporator at 40 °C to obtain yield viscous residues (20 g). The crude extract was kept in a tightly closed containers in a deep freezer till further analysis.

### 2.2. Gas Chromatography–Mass Spectrometry (GC-MS) Analysis of Psyllium Extract

The phytochemical constituents of PHEE were determined using Trace GC1300-TSQ mass spectrometer (Thermo Scientific, Austin, TX, USA) with a direct capillary column TG–5MS (30 m × 0.25 mm × 0.25 µm film thickness). The column temperature was initially held at 60 °C and then increased by 8 °C/min to 300 °C held for 2 min. Further, the temperature was increased to 310 °C by 20 °C/min and hold for 2 min. The injector and MS transfer line temperatures were maintained at 250 °C and 260 °C, respectively. Helium was used as a carrier gas at a constant flow rate of 1 mL/min. The solvent delay was 3 min, and diluted samples (1 µL) were injected automatically using Autosampler AS1300 coupled with GC in the split mode. EI mass spectra were collected at 70 eV ionization voltage over the range of m/z 50–650 in full scan mode, and the ion source temperature was set to 250 °C. The components were identified by comparing their retention times and mass spectra using the Wiley Spectral library search program [12].

### 2.3. Experimental Animals

Forty adult male albino rats (175–188 g) used in the experiment were kept in metal cages under standard hygienic conditions; temperature: 22 ± 2 °C, relative humidity; 40–60%, photoperiod; 12 h. dark: 12 h light. Rats were fed on a balanced pelleted diet and good water source was offered ad libitum. All procedures were in line with the university strategies for the care of experimental animals approved by the ZU-IACUC Committee of the Faculty of Veterinary Medicine, Zagazig University, Egypt (Approval number: ZU-IACUC/2/F/89/2020).

### 2.4. Experimental Protocol

After seven days of acclimatization, 40 adult male albino rats were allocated into four groups (10 rat/group): (1) the control group (CON); (2) Triton X-100 induced hyperlipidemic group—overnight fasting rats were intraperitoneally injected with a single dose of Triton X-100 (100 mg/kg body weight) on the 21st day of trial onset; (3) PHEE group—PHEE was orally administered (100 mg/kg body weight dissolved in 1 mL of distilled water) by gastric tube from the first day of the experiment until the fortieth day, once daily; (4) PHEE +Triton group—rats received PHEE orally with the induction of hyperlipidemia by intraperitoneal Triton X-100 injection by the same dose, route and time as in groups 2 and 3.

### 2.5. Collection of Blood Samples

At the terminus, blood samples were collected from the retro-orbital plexus of overnight fasting rats without anticoagulant in plain and clean centrifuge tubes (El Nasr Company, Egypt) to separate the serum by centrifugation at 3000 rpm for 15 min for biochemical study. The serum was kept at −20 °C for two days until the biochemical analysis.

### 2.6. Serum Lipid Profile

The total lipids, total cholesterol, triglyceride, and high-density lipoprotein (HDL-C) in serum were estimated according to Zöllner and Kirsch [13], Roeschlau, et al. [14], McGowan, et al. [15], and Young [16], respectively. Low-density lipoprotein (LDL-C) and very-low-density lipoprotein cholesterol (VLDL-C) were calculated by a mathematical relationship described by Friedewald, et al. [17]. The atherogenic index (AI) was calculated according to the mathematical relationship described by Khaza [18].

### 2.7. Pancreatic Function Tests, Serum Leptin, and Adiponectin Hormones

Serum glucose level was estimated according to Tietz [19]. Serum insulin level was estimated using THERMO scientific rat insulin ELISA Kit (Inchinnan, UK) according to Bates [17]. Serum glycated hemoglobin (HbA1c) was estimated using rat HbA1c ELISA Kit. Homeostatic model assessment for insulin resistance (HOMA-IR) was calculated according to Matthews, et al. [20]. Serum leptin and adiponectin levels were estimated using DRG ELISA Kit (DRG, Instrument, Gmbh, Marburg, Germany) according to Considine, Sinha, Heiman, Kriauciunas, Stephens, Nyce, Ohannesian, Marco, McKee and Bauer [21].

### 2.8. Liver Function Tests

The serum alanine aminotransferase (ALT) and aspartate aminotransferase (AST) activity were determined according to Reitman and Frankel [22]. The serum alkaline phosphatase activity was measured according to the modified Moss [23]. The serum gamma-glutamyl transferase was assessed using the kinetic colorimetric method of Szasz [24] and Bilirubin determined based on the method of Monnet [25].

The total serum protein and albumin levels were estimated according to Grant [26] and Doumas, et al. [27], respectively. According to Doumas and Biggs [28], the serum globulins levels were calculated by subtracting albumin values from total protein values. The albumin/globulin (A/G) ratio was calculated by dividing Albumin level by globulin level [29].

### 2.9. Serum Lipid Peroxidation and Antioxidant Enzyme Activity

Malondialdehyde (MDA) activity was determined as a marker for lipid peroxidation and oxidative stress, according to Ohkawa, et al. [30]. Glutathione peroxidase and catalase activity were determined according to Beutler [31] and Aebi [32], respectively.

### 2.10. Histopathological Examination

Specimens from the liver and pancreas of rats were dissected out and fixed in 10% neutral formalin. Paraffin segments of 5 µ thickness were organized from all specimens, stained by hematoxylin and eosin (H & E) [33], and observed microscopically.

### 2.11. Statistical Analysis

Data were analyzed using one-way analysis of variance (ANOVA) using the GLM procedure in SPSS (SPSS Inc., Chicago, IL, USA). Shapiro–Wilk’s test was used to verify the normality, and Levene’s test to verify homogeneity of variance components between experimental treatments. Duncan’s test was used to compare the differences between the means at 5% probability [34]. Variation in the data was expressed as mean ± SE, and the significance was set at *p* < 0.05.

## 3. Results

### 3.1. Gas Chromatography–Mass Spectrometry (GC-MS) Analysis

The gas chromatography, Mass spectroscopy (GC-MS) analysis of PHEE showed 17 distinct biological active constituents belonging to diverse natural element groups with different retention times that includes; DL-Arabinose 2,3,4,5-Tetrahydroxypentanal (27.21%); D-Xylose (13.80%); Ethyl 9.cis.,11. trans.-octadecadienoate (11.95%); 9,12-Octadecadienoic Acid, Methyl Ester, (E, E)-(11.86%); 9-Octadecenoic acid, methyl ester, (E)- (5.85%); Myristicin (4.15%); 9,12,15-Octadecatrienoic acid, 2,3-bis[(trimethylsilyl)oxy]propyl ester, (Z,Z,Z)-(3.35%); Hexadecanoic acid, methyl ester (2.94%); 2-Methoxy-4-Vinylphenol (2.91%); β-Sitosterol (2.57%); 7a,12a-Dihydroxy-cholestene-3-one (2.22%); Undecane (2.13%); Pentacosane (2.04%); Dodecamethyle cyclohexasiloxane (2.01%); Stigmata-4-en-3-one (1.91%); 3,7,11,15,18-Pentaoxa-2,19-disilaeicosane, 2,2,19,19-tetramethyl (1.89%); Ar-turmerone (1.20%). The recognized compounds, their molecular formula, and peak area (%) are shown in Table 1.

### 3.2. Effect on Lipid Profile

Triton-induced hyperlipidemic rats showed a rise in total lipids, TC, TG, LDL-c, VLDL-c, and AI serum levels than the CON group (*p* < 0.05). The elevated levels decreased in hyperlipidemic rats administered with PHEE (*p* < 0.05). The serum HDL-c level was reduced more in the hyperlipidemic rats than in the CON group (*p* < 0.05). The HDL-c level was elevated more in hyperlipidemic rats with PHEE and comparable to the hyperlipidemic group (*p* < 0.05). The PHEE group showed insignificant changes in the lipid profile compared to the CON group (*p* > 0.05) (Table 2).

### 3.3. Pancreatic Function Tests, Serum Leptin, and Adiponectin Hormones

Table 3 showed significant elevation in levels of glucose, insulin, HbA1c, HOMA IR index, and leptin hormone in triton-induced hyperlipidemic rats compared to the CON group (*p* < 0.05). In contrast, hyperlipidemic rats treated with PHEE showed significant diminution in these parameters compared to hyperlipidemic rats (*p* < 0.05). A significant decline was detected in adiponectin hormone in hyperlipidemic rats compared to the CON group, while the hyperlipidemic rats treated with PHEE showed a significant rise compared to the hyperlipidemic rats. The PHEE group revealed insignificant fluctuations compared to the CON group (*p* > 0.05).

### 3.4. Liver Functions Tests

Table 4 displayed more significant rises in serum activities of ALT, AST, and GGT in triton-induced hyperlipidemic rats than the CON group (*p* < 0.05). Conversely, hyperlipidemic rats treated with PHEE showed a more significant reduction in these parameters than triton-induced hyperlipidemic rats (*p* < 0.05). Moreover, the PHEE group revealed insignificant variations compared to the CON group (*p* > 0.05). Triton-induced hyperlipidemic rats showed hypoproteinemia, hypoalbuminemia, hypo-globulinemia, and a decrease in the A/G ratio compared to the CON group (*p* < 0.05). However, hyperlipidemic rats treated with PHEE showed a significant elevation in total proteins, albumin, globulins, and A/G ratio. Furthermore, the PHEE group revealed insignificant deviations compared with the CON group (*p* > 0.05).

### 3.5. Changes in Serum Lipid Peroxidation and Antioxidant Enzyme Activity

Table 5 revealed an increase in the serum MDA in hyperlipidemic rats than the CON group (*p* < 0.05). Conversely, hyperlipidemic rats exhibited a decrease in serum GSH and CAT compared to0 the CON group (*p* < 0.05). Hyperlipidemic rats treated with PHEE displayed a diminution in MDA level and an increase in serum GSH and CAT compared to hyperlipidemic rats (*p* < 0.05). However, the PHEE group showed insignificant changes in the aforementioned parameters compared to the CON group (*p* > 0.05).

### 3.6. Histopathological Finding

**Figure 1:**
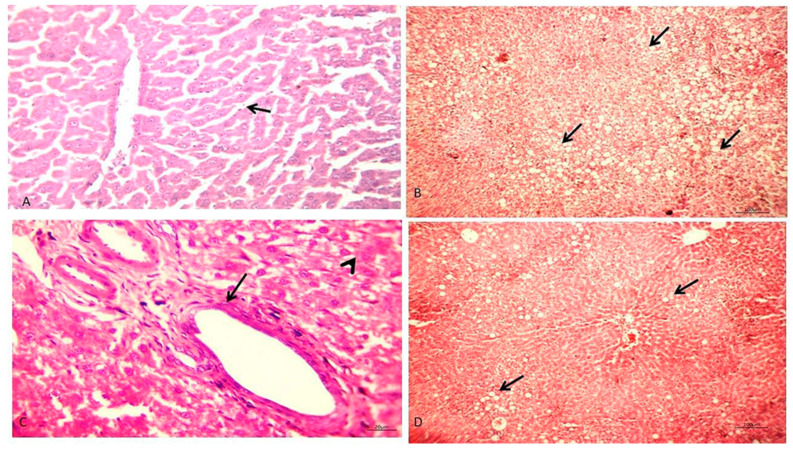
Photomicrograph of H & E stained liver section of different groups. (**A**) The liver of normal control rats showing normal hepatic parenchyma (arrow) (×400). (**B**) Hyperlipidemic rats showing intense peripherlobular microsteatosis (arrow) and acute cell swelling in the remaining hepatic cells (×100). (**C**) The liver of rats administered with psyllium extract showing only hyperplastic Kupfer cells (arrowhead) and mild fibroblastic proliferation in the portal area (arrow). (×400). (**D**) Liver of hyperlipidemic rats treated with psyllium extract showing mild microsteatosis (arrow) in peripherlobular patterns of the hepatic lobules and normal remaining hepatic cells. (×100).

The liver of CON rats showed a typical hepatic architecture (Figure 1A). The liver of triton-induced hyperlipidemic rats showed intense degenerative changes varying from acute cell swelling to microsteatosis, mainly involving the peripherlobular zones of most hepatic lobules. Moreover, large fatty droplets induce hepatocytes’ rupture with fat cyst formation within the hepatic parenchyma (Figure 1B). The PHEE group showed normal hepatic parenchyma with hyperplastic Kupfer cells, besides mild periductular fibroblasts in a few portal areas (Figure 1C). Hyperlipidemic rats treated with PHEE showed mild microsteatosis involving a few hepatocytes in peripherlobular hepatic parenchyma, and round cell aggregations were observed in a few portal areas with hyperplastic Kupfer cells (Figure 1D).

**Figure 2:**
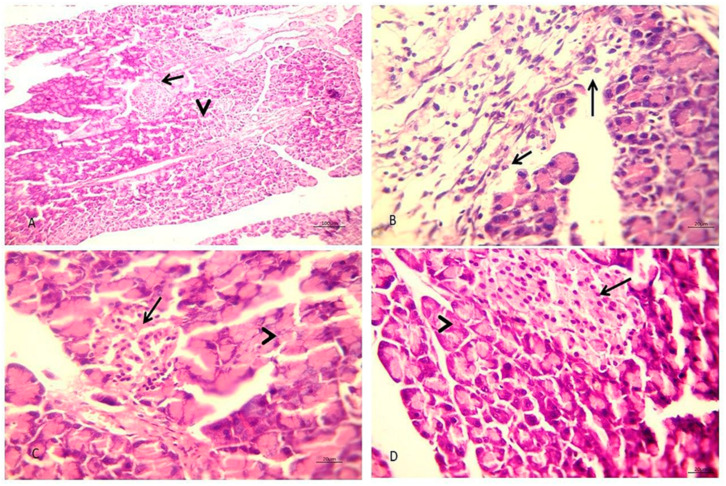
Photomicrograph of H & E stained pancreas section of different groups. (**A**) The pancreas of normal control rats showing normal endocrine (arrow) and exocrine portions (arrowhead) (×100). (**B**) The pancreas of hyperlipidemic rats showing focal replacement of pancreatic acini with edematous fibrous tissue containing round cells and granulocytes (arrow) (×400). (**C**) The pancreas of rats administered with psyllium extract only showing normal endocrine (arrow) and exocrine pancreas (arrowhead) (×400). (**D**) The pancreas of hyperlipidemic rats treated with Psyllium showing hypertrophied endocrine (arrow) and active exocrine (arrowhead) (×400).

CON rats showed normal endocrine and exocrine portions in pancreas histopathology (Figure 2A). The pancreas of triton-induced hyperlipidemic rats showed focal destruction, replacement by edematous fibrous tissue containing numerous inflammatory cells, and some acini with fatty droplets or lysis (Figure 2B). The PHEE group showed normal endocrine and exocrine pancreas and interstitial tissue (Figure 2C). Pancreatic sections from PHEE treated hyperlipidemic rats revealed hypertrophied endocrine pancreas with an active exocrine pancreas (Figure 2D).

## 4. Discussion

Hyperlipidemia is considered one of the common factors causing cardiovascular diseases, accounting for one-third of total deaths worldwide [35]. Nowadays, synthetic hyperlipidemic medications have diminished gradually due to their related side effects and drug resistance development. Therefore, the usage of medicinal plants has currently increased. The gas chromatography-mass spectroscopy (GC-MS) analysis of PHEE identified the presence of 17 engaging biological active constituents; DL-Arabinose 2,3,4,5-Tetrahydroxypentanal (27.21%); D-Xylose (13.80%); Ethyl 9.cis.,11. trans.-octadecadienoate (11.95%); 9,12-Octadecadienoic Acid, Methyl Ester, (E, E)-(11.86%); 9-Octadecenoic acid, methyl ester, (E)-(5.85%); Myristicin (4.15%); 9,12,15-Octadecatrienoic acid, 2,3-bis[(trimethylsilyl)oxy]propyl ester, (Z,Z,Z)-(3.35%); Hexadecanoic acid, methyl ester (2.94%); 2-Methoxy-4-Vinylphenol (2.91%); β-Sitosterol (2.57%); 7a,12a-Dihydroxy-cholestene-3-one (2.22%); Undecane (2.13%); Pentacosane (2.04%); Dodecamethyle cyclohexasiloxane (2.01%); Stigmata-4-en-3-one (1.91%); 3,7,11,15,18-Pentaoxa-2,19-disilaeicosane, 2,2,19,19-tetramethyl (1.89%); Ar-turmerone (1.20%).

The presence of 9,12,15-Octadecatrienoic acid, 2,3-bis[(trimethylsilyl)oxy]propyl ester, (Z,Z,Z)- was also identified with 0.98% in the methanol leaves extract of *Dendrophthoe falcata* Ettingsh Stem [36], with 2.41% in *Ficus Mollis* Vahl leaves [37], 4.5% in *Callyspongia Crassa* (porifera) extract [38], 0.3% in *Pleurotus cornucopiae* (Paulet) [39], 0.95% in the extract of *Sida cordata* (Burm. f.) [40]. The presence of 9,12-Octadecadienoic acid, methyl ester, (E,E)- was identified with 1.72% in Malaysian *Andrographis paniculata* leaf extract [41], 3.89% in *Sida cordata* (Burm.f.) extract [40], 6.62% *in Gynura segetum* leaf extracts [42], 10.9% in *Robinia pseudoacacia* L. seed extract [43]. The presence of 9-Octadecenoic acid, methyl ester, (E)-was identified with 4.6% in the ethanol extract of *Avicennia marina* leaves [44], 17.00% in the flower extract of moringa (Moringa oleifera), while the seed extracts were with 36.94% [45].

All rats injected with Triton displayed hyperlipidemia and showed dyslipidemia. Triton X-100 stops eliminating TG-rich lipoproteins and encourages acute hyperlipidemia in animal models [4]. The detected hypertriglyceridemia due to Triton X-100 injection increased VLDL emission by the liver, followed by a diminution in VLDL and LDL catabolism. The AI index was significantly elevated with increasing cholesterol, TG, and LDL-C and decreasing HDL-C. These findings were established by histopathological examination of the hyperlipidemic rats’ livers that displayed intense peripherlobular microsteatosis and acute cell swelling. Hyperlipidemic rats that received PHEE showed decreased lipogram levels and increased HDL-C in contrast to the hyperlipidemic group. These results are attributed to the arabinoxylans (arabinose and xylose) content of PHEE detected by GC-MS analysis. These ingredients bind with bile acids, making a complex that prevents the bile reabsorption from the small intestine; thus, stimulating the production and secretion of bile acids to replace the missing acid [46].

Therefore, cholesterol is extracted from the circulation to produce bile acids. Our results were confirmed with histopathological examination of rats’ livers that revealed mild microsteatosis in few hepatocytes in peripherlobular hepatic parenchyma. The hypolipidemic effect of PHEE may be attributed to its β-sitosterol and Ar-turmerone content. Yuanet al. [47] and Ling et al. [48] demonstrated the hypolipidemic effect of the β-sitosterol and antihyperlipidemic effect of the turmerone on hyperlipidemic rats.

Serum leptin concentration in triton-induced hyperlipidemic rats increased versus CON rats. Leptin is formed by adipose tissue as fat packing reserves in the body, and it adjusts food consumption and energy disbursement [49], thus, indicating that the elevated serum leptin levels detected are likely a consequence of the increased fat accumulation. The leptin level reduced in hyperlipidemic rats that received PHEE in contrast to hyperlipidemic rats. Leptin reduction is due to decreasing the fat mass in the body by the psyllium husk extract’s hypolipidemic effect, leading to attenuation of the proinflammatory environment accompanying hyperlipidemia [50]. The results of the serum adiponectin hormone in hyperlipidemic rats showed a significant decline in contrast to the CON. This result is because adiponectin concentration is conversely related to body fat mass [51]. Hyperlipidemic rats treated with PHEE showed an increase in adiponectin levels compared to hyperlipidemic rats. This increase is due to the PHEE hypolipidemic effect that reduces body fat accumulation and increases adiponectin [52].

Pancreatic function analysis showed a significant increase in serum glucose, insulin, HbA1c, and HOMA-IR in hyperlipidemic rats than the CON. These results may be due to high cholesterol accumulated in β-cells leading to lipotoxicity by reducing transcription factors expression required for β-cell [53]. Furthermore, hyperlipidemia is hazardous for insulin resistance development [54]. Hyperlipidemic rats administered with PHEE showed a diminution in serum glucose, insulin, HbA1c, and HOMA-IR compared to hyperlipidemic rats. These results may be due to arabinoxylans’ excellent fibrous contents (arabinose and xylose) in PHEE. The fibrous content can help control the body’s glycemic response to food, reducing insulin and blood glucose levels [55]. Moreover, the β-sitosterol component of PHEE has an antidiabetic effect [56]. Our results were confirmed by a histopathological examination that revealed hypertrophied endocrine pancreas with an active exocrine pancreas.

Liver function analysis showed a significant rise in ALT serum activities, AST, and GGT with a significant decline in serum total protein, albumin, globulin, and A/G ratio in hyperlipidemic rats than the CON group. These results may be due to hyperlipidemia induced by Triton that causes fat penetration in hepatocytes, leading to hepatocellular damage [57]. Moreover, hepatocytes’ extra fat accumulation may cause hepatocellular damage through the direct cellular cytotoxicity interceded by lipid peroxidation, free fatty acids (FFAs), mitochondrial impairment, oxidative stress, and cytokine-induced hepatotoxicity, ultimately leading to liver malfunction [58]. This also explains the significant reductions in serum total protein, albumin, globulin, and A/G ratio in triton-induced hyperlipidemic rats. These results were reinforced by histopathological findings, which revealed intensive degenerative changes varying from acute cell swelling to microsteatosis involving most hepatic lobules. Hyperlipidemic rats treated with PHEE showed a significant diminution in serum liver enzymes and significant elevation in proteinogram compared to the hyperlipidemic rats. These results may be due to the PHEE components (9,12-octadecadienoic acid, methyl ester, and β-sitosterol) which act as hepatoprotective agents [59,60]. The hepatoprotective effect of PHEE may also be attributed to myristicin, which is reported as having anti-inflammatory, hepatoprotective, and anticancer effects [61]

The present study showed a significant serum MDA flow and diminution in serum GSH and CAT in hyperlipidemic rats versus the CON group, which may be due to metabolic disorders caused by hyperlipidemia. This syndrome is characterized by several metabolic disorders resulting in free radicals’ formation and increased MDA levels. Triton x-100 increased oxidative stress through an elevation in Thio Barbituric Acid Reactive Substances (TBARS) accompanied by the reduction in glutathione and antioxidant activities demonstrating increased ROS production [62], consequently resulting in the depletion of GSH and CAT levels in hyperlipidemic conditions. Improvements were noticed in hyperlipidemic rats that received PHEE, representing a reduction in MDA activity while a significant rise in GSH and CAT levels than the hyperlipidemic group, concordant with a similar study [63]. These results may be attributed to the β-sitosterol content of PHEE having an antioxidant effect [56,60]. Additionally, the antioxidant effect of PHEE may be due to turmerone [48].

## 5. Conclusions

Taken together, our study investigated the efficacy of psyllium husk ethanolic extract (PHEE) on Triton X-100 induced hyperlipidemic rats by studying changes in hepatic and pancreatic function and histopathology. The results of this study indicated that psyllium husk ethanolic extract has potent effects against hyperlipidemia, hyperglycemia, and oxidative stress due to its high biological active constituents detected by GCMS. Psyllium husk ethanolic extract can reduce lipid peroxidation, increase CAT and GSH activities, and increase the hormones, leptin, and adiponectin associated with hyperlipidemia. The administration of PHEE relieved the histopathological changes in the liver and pancreas caused by hyperlipidemia. This study’s findings may help develop a novel trial against the effects of hyperlipidemia in the future. However, a limitation of this study is that it did not analyze the expression involved in glucose and lipid metabolism in hyperlipidemic rats. Moreover, we did not use different levels of PHEE to figure out the optimal level to counteract the side effects of hyperlipidemia. Therefore, it is recommended to consider the analysis of genes related to lipid and glucose metabolism and to evaluate varying levels of PHEE administration in the future.

## Figures and Tables

**Table 1:** The identified compounds of PHEE by GC-MS analysis.

No	Retention Time (min)	The Chemical Name of the Compound	Molecular Formula	Peak Area (%)
1	17.82	DL-Arabinose 2,3,4,5-Tetrahydroxypentanal	C5H10O5	27.21
2	29.21	D-Xylose	C5H10O5	13.80
3	29.10	Ethyl 9.cis.,11. trans.-octadecadienoate	C20H36O2	11.95
4	27.89	9,12-Octadecadienoic Acid, Methyl Ester, (E, E)	C19H34O2	11.86
5	28.01	9-Octadecenoic acid, methyl ester, (E)-	C19H36O2	5.85
6	26.06	Myristicin	C11H12O3	4.15
7	19.87	9,12,15-Octadecatrienoic acid, 2,3-bis [(trimethylsilyl)oxy]propyl ester, (Z,Z,Z)-	C27H52O4Si2	3.35
8	24.75	Hexadecanoic acid, methyl ester	C17H34O2	2.94
9	11.18	2-Methoxy-4-Vinylphenol	C9H10O2	2.91
10	19.47	β-Sitosterol	C29H50O	2.57
11	18.77	7a,12a-Dihydroxy-cholestene-3-one	C27H44O3	2.22
12	16.04	Undecane	C11H18O	2.13
13	29.67	Pentacosane	C25H52	2.04
14	18.90	Dodecamethyle cyclohexasiloxane	C12H36O6	2.01
15	19.30	Stigmata-4-en-3-one	C29H48O	1.91
16	18.01	3,7,11,15,18-Pentaoxa-2,19-disilaeicosane, 2,2,19,19-tetramethyl	C17H40O5	1.89
17	19.41	Ar-turmerone	C15H20O	1.20

**Table 2:** Changes in lipogram and atherogenic index (mean value ±SE).

	CON	Triton	PHEE	PHEE + Triton	F. Test
Total lipids (mg/dL)	624.60 ^c^ ± 0.30	750.40 ^a^ ± 0.67	608.80 ^c^ ± 0.91	690.80 ^b^ ± 0.38	**
Cholesterol(mg/dL)	180.40 ^c^ ± 0.61	266 ^a^ ± 0.40	177.60 ^c^ ± 0.22	202.20 ^b^ ± 0.44	**
TG (mg/dL)	110.20 ^c^ ± 0.47	164.40 ^a^ ± 0.50	109.80 ^c^ ± 0.54	115.40 ^b^ ± 0.61	**
HDL-c (mg/dL)	63.60 ^a^ ± 0.69	42 ^c^ ± 0.32	64 ^a^ ± 0.80	64.20 ^b^ ± 0.28	**
LDL-c (mg/dL)	94.76 ^c^ ± 0.67	191.12 ^a^ ± 0.18	91.64 ^c^ ± 0.42	109.32 ^b^ ± 0.72	**
VLDL-c (mg/dL)	22.04 ^c^ ± 0.49	32.88 ^a^ ± 0.99	21.96 ^c^ ± 1.10	23.08 ^b^ ± 0.52	**
Atherogenic index	0.239 ^c^ ± 0.013	0.600 ^a^ ± 0.035	0.235 ^c^ ± 0.032	0.254 ^b^ ± 0.017	**

^a, b, c^ Means within the same column having different superscript letters were significantly different. ** significant at *p* ≤ 0.01.

**Table 3:** Changes in some pancreatic function tests, serum leptin, and adiponectin hormones (mean value ± SE).

	CON	Triton	PHEE	PHEE + Triton	F. Test
Serum Glucose (mg/dL)	90.84 ^c^ ± 0.38	193 ^a^ ± 0.25	90.64 ^c^ ± 3.09	112.60 ^b^ ± 0.86	**
Insulin (nIU/mL)	11.68 ^c^ ± 0.34	22.12 ^a^ ± 0.94	10.07 ^c^ ± 0.89	16.10 ^b^ ± 1.75	**
Hb-A1-c (ng/mL)	1.60 ^c^ ± 0.03	6.19 ^a^ ± 0.35	1.53 ^c^ ± 0.10	3.04 ^b^ ± 0.35	**
Leptin (ng/mL)	2.01 ^c^ ± 0.09	10.05 ^a^ ± 0.71	2.06 ^c^ ± 0.21	4.12 ^b^ ± 0.60	**
HOMA-IR	2.60 ^c^ ± 0.07	10.56 ^a^ ± 0.68	2.38 ^c^ ± 0.14	4.56 ^b^ ± 0.63	**
Adiponectin (pg/mL)	53.53 ^a^ ± 1.6	27.76 ^c^ ± 1.7	55 ^a^ ± 0.18	41.23 ^b^ ± 1.69	**

^a, b, c^ Means within the same column having different superscript letters were significantly different. ** significant at *p* ≤ 0.01.

**Table 4:** Changes in liver function tests (mean value ±SE).

	CON	Triton	PHEE	PHEE + Triton	F. Test
ALT(U/l)	46.40 ^c^ ± 1.86	86.80 ^a^ ± 2.72	45.40 ^c^ ± 2.24	59.33 ^b^ ± 0.98	**
AST(U/l)	28.00 ^c^ ± 0.54	49.20 ^a^ ± 0.86	27.20 ^c^ ± 2.37	34.50 ^b^ ± 0.99	**
GGT(U/l)	25.80 ^c^ ± 1.52	52.20 ^a^ ± 2.08	28.80 ^c^ ± 2.35	40.33 ^b^ ± 2.43	**
Total protein (U/l)	7.28 ^a^ ± 0.09	3.94 ^c^ ± 0.14	7.41 ^a^ ± 0.14	6.31 ^b^ ± 0.18	**
Albumin (U/l)	3.89 ^a^ ± 0.09	1.78 ^c^ ± 0.10	4.11 ^a^ ± 0.10	3.29 ^b^ ± 0.14	**
Globulin (U/l)	3.39 ^a^ ± 0.12	2.16 ^c^ ± 0.15	3.29 ^a^ ± 0.23	3.02 ^b^ ± 0.22	**
A/G (U/l)	1.15 ^a^ ± 0.06	1.82 ^c^ ± 0.03	1.24 ^a^ ± 0.13	1.08 ^b^ ± 0.15	**

^a, b, c^ Means within the same column having different superscript letters were significantly different. ** significant at *p* ≤ 0.01.

**Table 5:** Changes in serum lipid peroxidation and antioxidant enzyme activity (mean value ± SE).

	CON	Triton	PHEE	PHEE + Triton	F. Test
MDA (nmol/mL)	11.40 ^c^ ± 0.92	27.00 ^a^ ± 1.64	11.40 ^c^ ± 1.36	17.00 ^b^ ± 1.92	**
GSH (mmol/l)	2.55 ^a^ ± 2.47	1.22 ^c^ ± 1.16	2.54 ^a^ ± 2.41	2.23 ^b^ ± 1.87	**
CAT U/l)	166.40 ^a^ ± 2.08	123.20 ^c^ ± 3.33	165.80 ^a^ ± 3.35	149.00 ^b^ ± 3.76	**

^a, b, c^ Means within the same column having different superscript letters were significantly different. ** significant at *p* ≤ 0.01.

## Data Availability

Data sharing not applicable.
